# Efficient Nickel Sulfide and Graphene Counter Electrodes Decorated with Silver Nanoparticles and Application in Dye-Sensitized Solar Cells

**DOI:** 10.1186/s11671-016-1456-z

**Published:** 2016-05-04

**Authors:** Gentian Yue, Fumin Li, Guang Yang, Weifeng Zhang

**Affiliations:** Key Laboratory of Photovoltaic Materials of Henan and School of Physics & Electronics, Henan University, Kaifeng, 475004 China

**Keywords:** Graphene, Nickel sulfide, Silver decorated, Counter electrode, Dye-sensitized solar cells

## Abstract

We reported a facile two-step electrochemical-chemical approach for in situ growth of nickel sulfide and graphene counter electrode (CE) decorated with silver nanoparticles (signed NiS/Gr-Ag) and served in dye-sensitized solar cells (DSSCs). Under optimum conditions, the DSSC achieved a remarkable power conversion efficiency of 8.36 % assembled with the NiS/Gr-Ag CE, much higher than that based on the Pt CE (7.76 %). The surface morphology of NiS/Gr-Ag CE exhibited a smooth surface with cross-growth of NiS, graphene, and Ag nanoparticles, which was beneficial to the fast mass transport of electrolytes; increased the contact area of electrolytes and active materials; and enabled to speed up the reduction of triiodide to iodide. The research on the electrochemical properties also showed that the NiS/Gr-Ag CE possessed lower charge transfer resistance and more excellent electrocatalytic activity in iodide/triiodide electrolyte compared to the Pt electrode.

**Electronic supplementary material:**

The online version of this article (doi:10.1186/s11671-016-1456-z) contains supplementary material, which is available to authorized users.

## Background

With photovoltaic technology being realized as a suitable renewable power for the fulfillment of increasing world energy consumption, dye-sensitized solar cells (DSSCs) have attracted a great deal of attention because of their potential as next-generation photovoltaic devices [1–3]. Up to the present, the highest photoelectric conversion efficiencies (PCEs) of DSSCs have achieved over 13 % [4]. In general, one DSSC consists of a dye-adsorbed nano-sized TiO_2_ anode, an iodide electrolyte, and a platinum (Pt) counter electrode (CE). Though Pt for the functional role fulfills requirements of CE, such as good transparency, excellent electrical conductivity, and electrocatalytic ability, researchers are still struggling to seek for low-cost substitutes for its high cost and scarcity [5–7]. In recent years, many interesting materials [8–18] have emerged as earth-abundant Pt replacements for DSSC CE catalysts including carbon materials, conducting polymers, transition metal sulfides, nitrides, carbides, and alloy. Within the group, nickel sulfide (NiS), as an electron collector and electrocatalyst in DSSCs, has aroused particular interest because of its low cost, superior electrocatalytic activity, and conductivity [19, 20]. The DSSC with NiS CE electrodeposited by a potential reversal technique showed comparable PCE (6.82 %) to the Pt-based cell (7.00 %) [21]. Ku et al. prepared a highly transparent NiS CE and presented good PCE for thiolate/disulfide-mediated DSSCs [22].

Moreover, the electrocatalytic ability and chemical activity of graphene were highly desirable for DSSCs; therefore, graphene and its composites (graphene and conducting polymers, sulfides, nitrides, and so on) were believed as an efficient route in enhancing the electrical and electrochemical performances of DSSCs [23–25]. Also, it is well known that silver (Ag) nanoparticles have been studied intensively for their potential applications in catalysis, biosensors, and environmental filtration for their high surface energy, excellent catalytic activity, and unique optical and electrical properties [26, 27].

Therefore, taking into account all of these factors, to search for more robust Pt-free CEs and improve DSSC performance, we prepared the nickel sulfide/graphene CE decorated with Ag nanoparticles (signed NiS/Gr-Ag) by using a facile two-step electrochemical-chemical approach for in situ growth and served in DSSCs. The extensively characterized DSSC with NiS/Gr-Ag CE was carried out and exhibited a considerably improved performance in PCE of 8.10 % under irradiation of 100 mW cm^−2^ (AM 1.5 G). The electrocatalytic ability of the NiS/Gr-Ag CE was also systematically investigated. This type of electrode was anticipated with many advantages including good electrical conductivity, easy electrolyte penetration, and high electrocatalytic ability.

## Methods

### Preparation of NiS/Gr-Ag CE

**Fig. 1:**
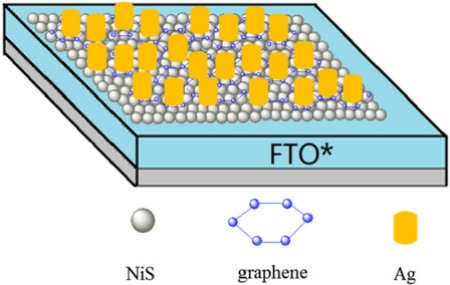
Schematic of NiS/Gr-Ag CE decorated with Ag nanoparticles

A NiS/Gr-Ag CE was prepared by a two-step electrochemical/chemical process and is outlined below. Firstly, a fluorine-doped tin oxide (FTO) glass (8 Ω cm^−2^, the thickness of 350 nm, Hartford Glass Co., USA) was modified by 4-aminothiophenol as we reported [28] and signed as FTO*. Secondly, the electrodeposition of NiS/Gr CE was carried out with an electrochemical analyzer system (CHI660E, Shanghai Chenhua Device Company, China). All experiments were implemented in a three-electrode cell, including a Pt foil as CE, an Ag/AgCl reference electrode, and FTO* (with an exposed area of 1 cm^2^) as the working electrode. The base electrodeposition solution consisted of 0.05 M nickel (II) chloride hexahydrate and 1.0 M thiourea in 50 ml deionized water and treated by ultrasonication for 30 min. The graphene flakes (UNI-ONWARD Corp., 99.9 %, Taiwan) were added into the base plating solution in weights ranging from 0, 5, 10, 15, and to 20 wt.%. The NiS/Gr CE sintered at 100 °C for 4 h in air, after being immersed in 50 ml ethanol solution, consisted of 2 ml thioglycolic acid and 0.2 g silver nitrate at 60 °C for 12 h and then sintered at 250 °C for another 30 min. The schematic of NiS/Gr-Ag CE is shown in Fig. 1. For comparison, the NiS, NiS/Gr, and NiS/Ag electrodes were prepared with a similar approach with NiS/Gr-Ag CE by using a three-electrode system; the Pt CE was prepared by soaking the FTO* substrates in 0.01 M H_2_PtCl_6_ ethanol solution; the graphene powder was mixed with a 1 wt.% solution of polyvinylidene fluoride in terpineol by a three-roll miller, and then the mixture was coated on the FTO* glass substrate by a doctor blade and dried at 200 °C for 30 min to get a Gr CE.

### Fabrication of DSSC

The multilayer TiO_2_ photoanode was prepared as described previously [29, 30]. The dye was loaded by soaking the TiO_2_ anode in 0.3 mM of dye Z-907 (Switzerland) ethanol solution for 12 h. Thus, the dye-sensitized TiO_2_ anode with thickness of 8–10 μm was obtained. The liquid electrolyte consisted of 0.05 M of I_2_, 0.1 M of LiI, 0.6 M of tetrabutylammonium iodide, and 0.5 M of 4-tert-butyl-pyridine in acetonitrile. The active area of the dye-coated TiO_2_ film was 0.4 × 0.7 cm^2^.

### Characterization

The surface morphology of the samples was observed by using a JSM-7001F field emission scanning electron microscope (SEM). Electrochemical impedance spectroscopy (EIS) measurements of the DSSC-based EIS were carried out under the simulating open-circuit conditions at ambient atmosphere, sealing with thermoplastic hot-melt Surlyn and leaving an exposed area of 1.0 cm^2^. The frequency of the applied sinusoidal AC voltage signal was varied from 0.1 to 10^5^ Hz, and the corresponding amplitude was kept at 5 mV in all cases. The incident photon-to-current conversion efficiency (IPCE) was conducted using a Newport lamp (Oriel 300 W xenon arc lamp) and light filters as a monochromatic light source and Si photovoltaic cell as reference. The photovoltaic test of DSSC was carried out by measuring the photocurrent-photovoltage (*J*-*V*) character curve under white light irradiation of 100 mW cm^−2^ (AM 1.5 G) from a solar simulator (XQ-500 W, Shanghai Photoelectricity Device Company, China) in ambient atmosphere.

## Results and Discussion

**Fig. 2:**
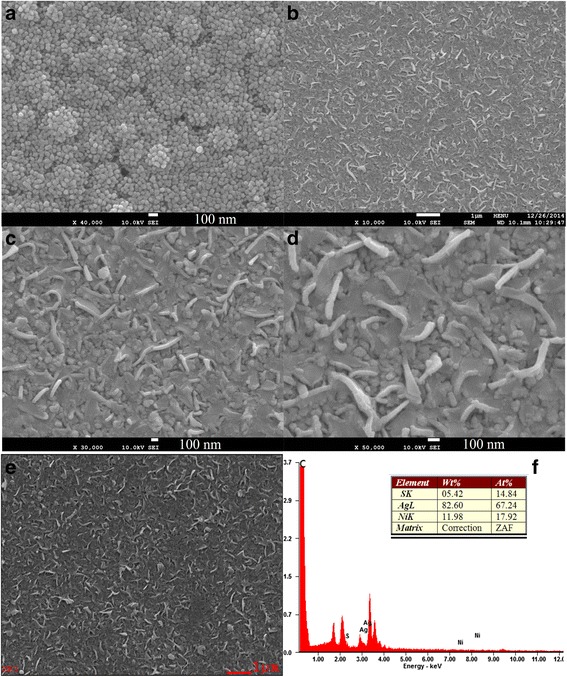
The SEM images of NiS CE (**a**), NiS/Gr-Ag CEs (**b**, **c**, **d**, **e**), and EDS of NiS/Gr-Ag CE (**f**)

Figure 2a shows the SEM image of NiS CE, which exhibited the NiS nanoparticles uniformly coated on the FTO* substrate and a few of pores formed on its surface. Figure 2b, c, d, and e presented the images of the NiS/Gr-Ag CE at low and high magnifications. From the three images, it is clear that the NiS/Gr-Ag CE possessed a uniform and smooth surface with graphene, NiS, and Ag nanoparticles distributed on the FTO* surface, which featured a nanowall network-like shape. We attributed the formation of the NiS/Gr-Ag nanowall to the high surface energy of Ag nanoparticles which made the Ag nanoparticles easily clustered and coated on the NiS/Gr surface. Such a distinctive nanowall network structure was conducive to storage and penetration of electrolytes, provided a large contact area for the electrolytes and active materials, and enabled to speed up the reduction of triiodide to iodide.

To check the composition of NiS/Gr-Ag CE, energy-dispersive spectrometry (EDS) was carried out. Figure 2f revealed that the presence of C, S, Ag, and Ni elements and strong signals were found in NiS/Gr-Ag CE. This illustrated that NiS was successfully deposited on the FTO* substrate. The strong signals for the Ag element as shown in Fig. 2f indicated that the Ag nanoparticles were filled in the pores and surface of NiS/Gr CE. The large amount of C element was responsible for the introduction of graphene flakes into the NiS/Gr-Ag CE. Consequently, the results could prove effectively that the NiS/Gr-Ag CE was successfully prepared.

**Fig. 3:**
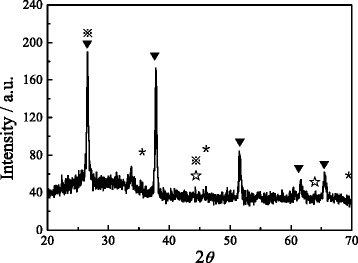
GIXRD patterns of NiS/Gr-Ag CE

To further confirm the composition of the NiS/Gr-Ag CE, XRD and Raman analyses were carried out as shown in Fig. 3 and Additional file 1: Figure S1. According to the Joint Committee on Powder Diffraction Standards (JCPDS card no. 3-1149), five very strong diffraction peaks at around 26.6°, 37.8°, 51.6°, 61.6° ,and 65.6° were corresponding to the signals of FTO and the diffraction peaks marked ※, ✰, and * were the contribution of graphene flakes (26.4° and 44.3°), Ag nanoparticles (44.1° and 64.1°), and NiS (35.5°, 45.8, and 69.4°), respectively. The Raman spectroscopy also revealed that the strong characteristic peaks of graphene were observed at 1350, 1579, and 2684 cm^−1^, respectively. Consequently, considering the results of EDS, XRD, and Raman spectra, the NiS/Gr-Ag CE has been synthesized successfully.

**Fig. 4:**
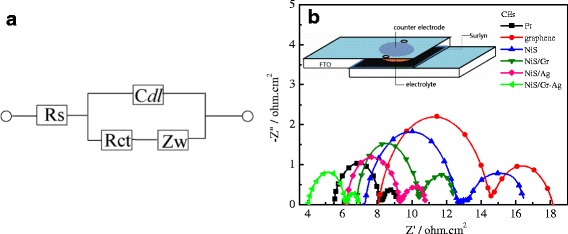
Equivalent circuits used for simulating the obtained EIS results (**a**). Nyquist plots of the symmetrical Pt, Gr, NiS, NiS/Gr, NiS/Ag, and NiS/Gr-Ag CEs for *I* ^−^/*I*_3_^−^ redox couple (**b**)

Figure 4 presents the Nyquist plots of Pt, graphene, NiS, NiS/Gr, NiS/Ag, and NiS/Gr-Ag CEs, and their corresponding parameters are listed in Table 1. *R*_s_ was the resistance value at the onset point of the first semicircle, and the *R*_*ct*_ was the radius of the first semicircle, and the semicircle at a low frequency represented the Nernst diffusion impedance (*Z*_*w*_) corresponding to the diffusion resistance of the *I*^−^/*I*_3_^−^ redox species. To our knowledge, *R*_s_ and *R*_ct_ were two crucial parameters for comparing the electrocatalytic abilities of various CEs, which were inversely correlated with the electrocatalytic ability of the CEs [31]. It is clear from Fig. 4 and Table 1 that the *R*_s_ for the Pt, graphene, NiS, NiS/Gr, NiS/Ag, and NiS/Gr-Ag CEs were 5.50 ± 0.02, 8.10 ± 0.02, 7.33 ± 0.02, 6.83 ± 0.02, 6.19 ± 0.02, and 4.05 ± 0.02 Ω cm^2^, respectively. The NiS/Gr-Ag CE possessed much lower *R*_s_ than the others, indicating a better electrical conductivity and electrocatalytic ability. The *R*_ct_ of Pt, graphene, NiS, NiS/Gr, and NiS/Ag CEs were 2.78 ± 0.02, 6.59 ± 0.02, 5.45 ± 0.02, 3.65 ± 0.02, and 3.18 ± 0.02 Ω cm^2^, respectively, following the orders of graphene CE > NiS CE > NiS/Gr CE > NiS/Ag CE > Pt CE. The NiS/Gr-Ag CE showed the smallest *R*_ct_ than that of the abovementioned CEs, but very close to that of the Pt CE. This meant that the NiS/Gr-Ag CE can rapidly reduce *I*_3_^−^ to *I*^−^ to speed up the diffusion of *I*_3_^−^ as effectively as Pt CE. The *Z*_*w*_ values obeyed the similar orders of NiS CE > graphene CE > NiS/Gr CE > NiS/Ag CE > Pt CE > NiS/Gr-Ag CE. However, in comparison with the NiS electrode, the graphene CE had a little smaller *Z*_*w*_ for its high conductivity. In a word, the NiS/Gr-Ag CE was provided with the least overpotential and most excellent electrochemical catalytic ability for an electron transferring in *I*^−^/*I*_3_^−^ redox species among the six CEs.

**Table 1:** Electrochemical parameters made from the impedance spectra and CVs of various CEs

Electrodes	*R*_*s*_ (Ω cm^−2^)	*R*_ct_ (Ω cm^−2^)	*Z*_*w*_ (Ω cm^−2^)	*J*_0_ (mA cm^−2^)	|*I*_pc_| (mA cm^−2^)	|*V*_pc_| (mV)	|*E*_pp_| (mV)
Pt	5.50 ± 0.02	2.78 ± 0.02	0.88 ± 0.02	1.35 ± 0.01	3.18 ± 0.02	0.16 ± 0.01	0.27 ± 0.01
Graphene	8.10 ± 0.02	6.59 ± 0.02	3.44 ± 0.02	0.96 ± 0.01	2.13 ± 0.02	0.25 ± 0.01	0.42 ± 0.01
NiS	7.33 ± 0.02	5.45 ± 0.02	3.76 ± 0.02	1.05 ± 0.01	2.46 ± 0.02	0.24 ± 0.01	0.39 ± 0.01
NiS/Gr	6.83 ± 0.02	3.65 ± 0.02	2.01 ± 0.02	1.11 ± 0.01	2.71 ± 0.02	0.20 ± 0.01	0.33 ± 0.01
NiS/Ag	6.19 ± 0.02	3.18 ± 0.02	1.49 ± 0.02	1.19 ± 0.01	2.95 ± 0.02	0.19 ± 0.01	0.32 ± 0.01
NiS/Gr-Ag	4.05 ± 0.02	2.17 ± 0.02	0.70 ± 0.02	1.88 ± 0.01	3.43 ± 0.02	0.17 ± 0.01	0.25 ± 0.01

**Fig. 5:**
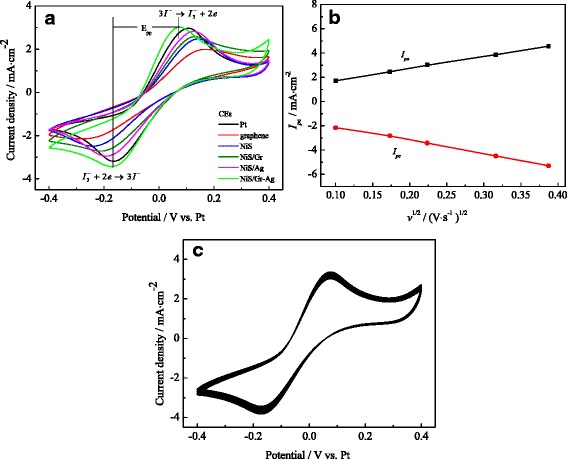
CVs for the Pt, Gr, NiS, NiS/Gr, NiS/Ag, and NiS/Gr-Ag CEs for *I* ^−^/*I*_3_^−^ redox couple (**a**). Relationship between the anodic and cathodic peaks current versus square root of scan rate (**b**). Fifty cycles of CVs of the NiS/Gr-Ag electrode at a scan rate of 50 mV s^−1^ (**c**)

Figure 5a depicted the cyclic voltammograms of the various electrodes under the *I*^−^/*I*_3_^−^ electrolyte system at a scan rate of 50 mV s^−1^. The peak to peak separation (*E*_pp_), the cathodic current density (*I*_pc_), and potential (*V*_pc_) were crucial parameters for comparing the electrocatalytic abilities of CEs [32, 33]. Among them, the |*I*_pc_| was positively correlated with the catalytic ability of CEs and the |*V*_pc_| and |*E*_pp_| were inversely correlated with the electrocatalytic activity of the CEs. From Fig. 5a, the NiS/Gr-Ag CE showed much higher |*I*_pc_| (3.43 ± 0.02 mA cm^−2^) than the Gr, NiS, NiS/Gr and NiS/Ag electrodes, a little higher than the Pt CE (3.18 ± 0.02 mA cm^−2^), indicating that the NiS/Gr-Ag CE effectively acted as a catalyst in the reaction of the *I*^−^/*I*_3_^−^ electrolyte, which attributed to the large active surface area and their synergistic catalytic effect of NiS (with superior electrocatalytic activity) and graphene (with high conductivity). The |*V*_pc_| of the CEs increased in the order of Pt CE (0.16 ± 0.01 V) < NiS/Gr-Ag CE (0.17 ± 0.01 V) < NiS/Ag CE (0.19 ± 0.01 V) < NiS/Gr CE (0.20 ± 0.01 V) < NiS CE (0.24 ± 0.01 V) < graphene CE (0.25 ± 0.01 V), while the |*E*_pp_| obeyed the order of NiS/Gr-Ag CE (0.25 ± 0.01 V) < Pt CE (0.27 ± 0.01 V) < NiS/Ag CE (0.32 ± 0.01 V) < NiS/Gr CE (0.33 ± 0.01 V) < NiS CE (0.39 ± 0.01 V) < graphene CE (0.42 ± 0.01 V). Interestingly, the NiS/Gr-Ag CE showed larger |*V*_pc_| but much higher |*I*_pc_| and smaller |*E*_pp_| compared to the Pt CE. This was responsible for the incorporated Ag nanoparticles with good electrical conductivity. In the case of NiS/Gr-Ag CE, Ag nanoparticles evenly coated on the NiS/Gr surface and still basically followed the morphology of the conductive graphene network, which would effectively facilitate transport of the electron and diffusion of redox electrolyte.

Furthermore, the nearly linear relationship can be found for the NiS/Gr-Ag CE for the relationship between the anodic current density (*I*_pa_), *I*_pc_, and the square root of the scan rate (*v*) as exhibited in Fig. 5b. This can be attributed to the introduction of Ag nanoparticles and graphene flakes, which was advantageous to the electron transport effectively for the *I*_3_^−^ reduction at the electrolyte|NiS/Gr-Ag CE interface, even at high scan rates. The results revealed that the reaction of *I*^−^/*I*_3_^−^ redox couple at CE was dominated by the diffusion-controlled transport and there was no specific interaction between *I*^−^/*I*_3_^−^ redox couple and NiS/Gr-Ag CE [34, 35].

The diffusion coefficients (*D*_*n*_) of *I*_3_^−^ in electrolytes of NiS/Gr-Ag and Pt CEs were also estimated of 3.30 × 10^−6^ and 2.4 × 10^−6^ cm^−2^ s^−1^ in the light of the Randles-Sevcik equation [36]. The *D*_*n*_ of NiS/Gr-Ag CE was much larger than that of the Pt CE, which can be attributed to the high conductivity for the graphene flakes, excellent electrocatalytic ability for the Ag nanoclusters, and the abundant pores in the NiS surface. Therefore, that was able to shorten the ion diffusion path and promote ion diffusion flux.

1 $$ {I}_{\mathrm{p}c}=K{n}^{1.5} AC{\left({D}_n\right)}^{0.5}{v}^{0.5} $$

where *K* was the constant of 2.69 × 10^5^; *n* meant the number of electrodes contributing the charge transfer; *A* was the area of the CE; and *C* and *v* represented the concentration of *I*_3_^−^ species and the scan rate, respectively.

The long-term electrochemical stability for the CE was very important to the performance of the device. As presented in Fig. 5c, the cyclic voltammetry (CVs) and the normalized cathodic and anodic peak current densities remained scarcely changed after being tested for 50 consecutive cycles, suggesting that the NiS/Gr-Ag electrode was provided with the characteristics of reversible redox activity, strong adhesiveness on the FTO* substrate, excellent electrochemical properties, and chemical stability [37].

**Fig. 6:**
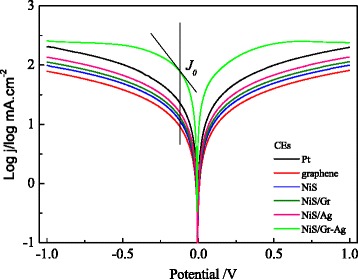
Tafel curves of the symmetrical Pt, graphene, NiS, NiS/Gr, NiS/Ag, and NiS/Gr-Ag CEs for *I* ^−^/*I*_3_^−^ redox couple

To further verify the results of the EIS, Fig. 6 displays the Tafel polarization curves of the Pt, graphene, NiS, NiS/Gr, NiS/Ag, and NiS/Gr-Ag CEs. The tangent slope of Tafel curves provided the information regarding the exchange current density (*J*_0_) [38]. The *J*_0_ for the different CEs were also estimated and summarized in Table 1. Compared to the graphene, NiS, NiS/Gr, and NiS/Ag CEs, the NiS/Gr-Ag CE possessed a remarkably enhanced *J*_0_, which was even slightly higher than that of the Pt CE. This meant that the NiS/Gr-Ag CE can trigger the reduction of *I*_3_^−^ to *I*^−^ more effectively than the others. The *R*_ct_ was inversely dependent on *J*_0_ and can be calculated based on Eqn. (2) [39]. The variance tendencies of the *R*_ct_ values calculated from the *J*_0_ values for the Pt, graphene, NiS, NiS/Gr, NiS/Ag, and NiS/Gr-Ag CEs were in accordance with those obtained in the EIS measurements. It may be concluded that NiS/Gr-Ag CE was more effective than the Pt CE in electrocatalyzing the *I*_3_^−^ reduction for DSSC. Furthermore, the limiting diffusion current density (*J*_lim_), obtained from the intersection of the cathodic branch with the *Y*-axis, was associated with the diffusion coefficient (*D*_*f*_) on the basis of Eqn. (3) [40]. For the different CEs, the changed tendencies of estimated *D*_*f*_ were generally in agreement with the variation of *D*_*n*_ obtained from the CV measurements. This result well confirmed that the NiS/Gr-Ag CE possessed excellent ion diffusivity.

2 $$ {J}_0=\frac{RT}{nF{R}_{ct}} $$

3 $$ {D}_f=\frac{l}{2\mathrm{n}\mathrm{F}\mathrm{C}}{J}_{\lim } $$

where *R* was the gas constant, *T* was the temperature, *F* was the Faraday constant, and *l* was the spacer thickness.

**Fig. 7:**
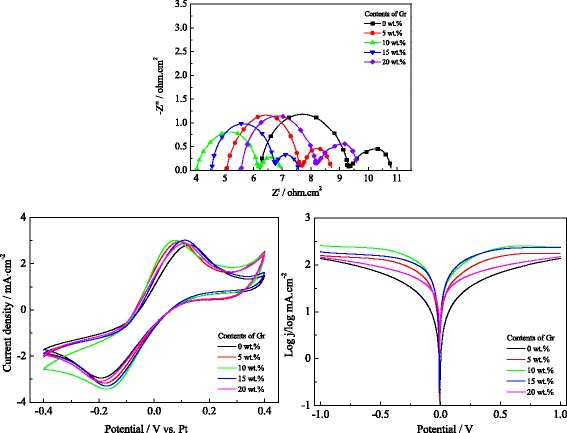
The influences of graphene contents on the electrochemical properties of counter electrodes

To further research the electrocatalytic ability and the effect of graphene contents on NiS/Gr-Ag CE, Fig. 7 gives the EIS, Tafel, and CVs of the samples with various graphene contents. With the weight ratio of graphene increasing in the base plating solution from 0 to 5 wt.%, the *R*_s_, *R*_ct_, |*E*_pp_|, and |*V*_pc_| values decreased and the *J*_0_ and |*I*_pc_| values shifted higher. The NiS/Gr-Ag CE achieved the most excellent electrochemical performance with 10 % of graphene. The possible reasons behind the performance with significant enhancements for the NiS/Gr-Ag CE were due to its large catalytic surface area and excellent conductivity of graphene and the synergetic effect of NiS, Ag, and graphene. As we know, the perfectly crystalline basal plane of a carbon material had very little catalytic effect [41]. Thus, the aggressive chemical treatments during the process of the electrodeposition also can possibly introduce significant oxygen-containing functional groups and provide the increased active sites for *I*_3_^−^ reduction to further boost the electrocatalytic ability of the samples [42]. While further increasing the graphene content to 15 or 20 wt.%, it resulted in a negative effect on the electrochemical performance of the samples made from the EIS, Tafel, and CVs analyses, which may be responsible for the poor electrocatalytic activity of graphene. Thus, the results indicated that the NiS/Gr-Ag CE with the suitable graphene can remarkably improve conductivity and electrocatalytic ability for *I*_3_^−^ reduction.

**Fig. 8:**
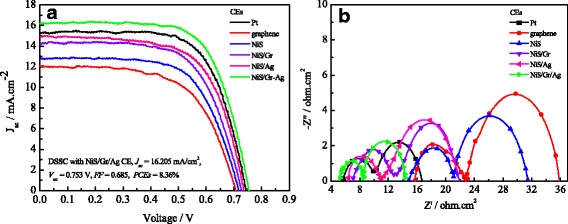
*J*-*V* characteristics (**a**) and Nyquist plots (**b**) of the DSSCs with various CEs for *I* ^−^/*I*_3_^−^ redox couple

The photovoltaic performance of DSSCs assembled with various CEs including Pt, graphene, NiS, NiS/Gr, NiS/Ag, and NiS/Gr-Ag CEs (signed DSSC 1, DSSC 2, DSSC 3, DSSC 4, DSSC 5, DSSC 6) were evaluated under the illumination of 100 mW cm^−2^. According to Table 2 and Fig. 8a, the photovoltaic parameters of the DSSCs with various CEs followed the order of DSSC 6 > DSSC 1 > DSSC 5 > DSSC 4 > DSSC 3 > DSSC 2 except for the fill factor (FF). Among the DSSCs, the ones assembled with the composite CEs including NiS/Gr, NiS/Ag, and NiS/Gr-Ag all exhibited better PCEs compared to those of the DSSC based on the graphene or NiS CE. When the NiS or NiS/Gr CE was decorated with Ag nanoparticles, the FF, the short-circuit current density (*J*_sc_), and PCE all showed remarkable improvement. Thus, the DSSC using NiS/Gr-Ag CE generated an enhanced PCE of 8.36 %, which was much higher than that of the Pt-based (7.76 %) and NiS/Ag-based (7.17 %) DSSCs. The reasons of the enhancement on PCEs for the DSSC with NiS/Gr-Ag CE can be ascribed to the improved FF and *J*_sc_ values, which was derived from the increased total current of *I*^−^/*I*_3_^−^ redox reaction, and have been proven by its large cathodic current density and enhanced diffusivity of *I*^−^/*I*_3_^−^ redox species, as indicated in the aforementioned CV tests. The improvement of FF possibly resulted from the decrease in the sum of *R*_s_ and *R*_ct_.

**Table 2:** EIS parameters and photoelectric properties of DSSCs with various CEs

Electrodes	DSSCs	*V*_oc_ (V)	*J*_sc_ (mA cm^−2^)	FF	PCEs (%)	*R*_s_ (Ω cm^−2^)	*R*_ct1_ (Ω cm^−2^)	*R*_ct2_ (Ω cm^−2^)	*C*_*μ*_ (μF)	*τ*_*n*(EIS)_ (ms)
Pt	1	0.743	15.289	0.683	7.76	5.72 ± 0.02	4.30 ± 0.02	6.61 ± 0.02	1084	7.165
Graphene	2	0.706	12.085	0.612	5.22	15.73 ± 0.02	7.05 ± 0.02	13.11 ± 0.02	404	5.296
NiS	3	0.717	12.836	0.651	5.99	14.88 ± 0.02	6.26 ± 0.02	10.37 ± 0.02	535	5.548
NiS/Gr	4	0.727	14.244	0.678	7.02	7.10 ± 0.02	5.90 ± 0.02	8.94 ± 0.02	696	6.222
NiS/Ag	5	0.735	14.909	0.654	7.17	6.46 ± 0.02	4.51 ± 0.02	10.97 ± 0.02	678	7.438
NiS/Gr-Ag	6	0.753	16.205	0.685	8.36	5.24 ± 0.02	3.24 ± 0.02	5.81 ± 0.02	1252	7.274

To better understand the dynamics of electron transport and recombination, EIS were carried out for the DSSCs with various CEs under an illumination of 100 mW cm^−2^. The *R*_ct1_ meant the interfacial charge transfer resistance at the CE and electrolyte; the *R*_ct2_ indicated the interfacial charge transfer resistance at the dye-sensitized photoanode and the electrolyte, and C_μ_ was the constant phase element resulting from the capacitor components in DSSC [43]. Figure 8b shows the EIS made from DSSCs 1 to 6. The series resistance (*R*_s_) were 5.72 ± 0.02, 15.73 ± 0.02, 14.88 ± 0.02, 7.10 ± 0.02, 6.46 ± 0.02, and 5.24 ± 0.02 Ω cm^2^, and the *R*_ct1_ were 4.30 ± 0.02, 7.05 ± 0.02, 6.26 ± 0.02, 5.90 ± 0.02, 4.51 ± 0.02, and 3.24 ± 0.02 Ω cm^2^ for the DSSCs from 1 to 6. The lowest *R*_ct1_ was noted at 3.24 ± 0.02 Ω cm^2^ for DSSC 6 due to its high active surface area and good catalytic property certified by CVs, Tafel, and EIS of the NiS/Gr-Ag CE. The tendency of *R*_s_ and *R*_ct1_ well agreed with the *J*_sc_ and open-circuit voltage (*V*_oc_) in Table 2, which reflected in the improvement of *V*_oc_ and PCE in DSSCs. In comparison with DSSCs from 1 to 5, DSSC 6 showed the smallest *R*_ct2_ of 5.24 ± 0.02 Ω cm^2^, suggesting that it had the lowest recombination rate. According to the equation *τ*_*n*(*EIS*)_ = *R*_*ct*2_ × *C*_*μ*_, the electron lifetimes (*τ*_*n*_) were calculated to be 7.165, 5.296, 5.548, 6.222, and 7.274 ms for DSSCs 1, 2, 3, 4, and 6, respectively. The longest electron lifetime for DSSC 6 indicated more effective suppression of the back reaction between a photoanode in the conduction band of TiO_2_ and *I*_3_^−^ in the electrolyte and also reflected in the enhancement of *J*_sc_, *V*_oc_, and PCE for DSSC 6. The reasons for the lowest recombination rate and longest electron lifetime of DSSC 6 perhaps ascribed to the excellent electrocatalytic ability and large contact areas between CEs and TiO_2_ photoanode that there would be less recombination for the generated electrons occurring at the interface of TiO_2_ nanocrystals|*I*^−^/*I*_3_^−^ electrolyte. However, despite the larger *τ*_*n*_ for the DSSC 5 than for the DSSC 6, DSSC 5 exhibited lower *J*_sc_, *V*_oc_, and PCE than DSSC 6 for its lager *R*_ct1_ and *R*_ct2_, which did not facilitate the charge transport interior of DSSC. As a consequence, compared with DSSCs 1 to 5, DSSC 6 showed lower recombination rate and longer electron lifetime than the others. Thus, DSSC 6 obtained the highest values of *J*_sc_ and PCE.

**Fig. 9:**
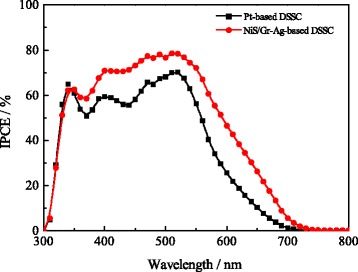
IPCE spectra of the DSSCs with the Pt and NiS/Gr-Ag CEs

Figure 9 shows the IPCE of DSSCs assembled with the NiS/Gr-Ag and Pt CEs, which displayed similar photoelectric responses at the range of 343 nm for the direct band gap photoelectron excitation of TiO_2_ [44]. Both DSSCs exhibited the highest quantum efficiency of 79.2 and 70.1 %, respectively, at 520 nm. The remarkable improvement for the photoelectric performance of DSSC with NiS/Gr-Ag CE attributed to the synergistic effect of NiS, graphene, and Ag nanoparticles, and this promoted the light capture of dye in this region and perhaps resulted in a higher *J*_sc_ and lower *R*_ct_ than that of DSSC with the Pt CE. This result was also in good agreement with diffuse reflection results analyzed above.

## Conclusions

In summary, NiS/Gr-Ag CE has been successful in situ grown on the surface of conductive glass through a facial two-step electrochemical-chemical approach and acted as Pt-free CE in DSSCs without any post-treatments. Under optimum conditions, the NiS/Gr-Ag CE exhibited amazing electrocatalytic activity and low charge transfer resistance for the reduction of *I*_3_^−^ and the DSSC with it produced a higher short-circuit photocurrent and power conversion efficiency (16.205 mA cm^−2^ and 8.36 %, respectively) than the DSSC with a Pt CE (15.289 mA cm^−2^ and 7.76 %, respectively). In consideration of this facile approach, efficient and low-cost NiS/Gr-Ag CE has a vast potential in scalable production of DSSCs.

## Additional file

Additional file 1: Figure S1. Raman spectra of the NiS/Gr-Ag CE.
