# Supplementary material for: Efficient Nickel Sulfide and Graphene Counter Electrodes Decorated with Silver Nanoparticles and Application in Dye-Sensitized Solar Cells
Source: Nanoscale Res Lett. 2016 May 4;11:239. doi: 10.1186/s11671-016-1456-z (PMC4854852; doi:10.1186/s11671-016-1456-z)
Supplement: Additional file 1: Figure S1. — Raman spectra of the NiS/Gr-Ag CE. [file 11671_2016_1456_MOESM1_ESM.doc]

**Additional file 1**

**Nickel Sulfide and Graphene Efficient Counter Electrode with Silver Nanoparticles Decorated and Application in Dye-Sensitized Solar Cells**

Gentian Yue, ^*,†^Fumin Li,^†^ Guang Yang,^†^ Weifeng Zhang^†^

^†^ *Key Laboratory of Photovoltaic Materials of Henan and School of Physics & Electronics, Henan University, Kaifeng 475004, China*

**Figure. S1** Raman spectra of the NiS/Gr-Ag CE.
